# Management Concepts of Bisphosphonate-Related Atypical Femoral Fractures

**DOI:** 10.3390/jcm14082858

**Published:** 2025-04-21

**Authors:** Liviu-Coriolan Misca, Rehan Gul, Jenel Marian Patrascu

**Affiliations:** 1 Orthopedics Department, University of Medicine and Pharmacy Victor Babes, Timisoara, 300041 Timișoara, Romania; patrascu.jenel@umft.ro; 2 Department Orthopaedics and Traumatology, Cork University Hospital, T12DC4A Cork, Ireland; 3 Department Orthopaedics and Traumatology, University College Cork, T12 K8AF Cork, Ireland; 4 Orthopedics Clinic II, Timiş County Emergency Clinical Hospital, 300723 Timisoara, Romania

**Keywords:** bisphosphonates, atypical femoral fractures (AFFs), cephalomedullary nailing (CMN), teriparatide

## Abstract

**Background/Objectives**: Osteoporosis is an important health issue worldwide, and bisphosphonates are commonly prescribed for its treatment. However, certain complications can occur with long-term bisphosphonate therapy. The complication highlighted in this study was atypical femoral fractures, which are rare but significant. The orthopedic consensus identifies surgical intervention as the gold-standard treatment for atypical femoral fractures, typically involving intramedullary or cephalomedullary nailing (CMN). The aim was to monitor patients for a follow-up period exceeding six months after surgical fixation with a CMN, with the majority of patients being followed up for more than 18 months after their initial surgery. **Methods**: This single-center analysis was conducted on a mixed cohort comprising a total of 10 patients. The study was conducted between September and November 2024. The inclusion criterion was surgical treatment for bisphosphonate-related atypical femoral fractures (AFFs) between June 2022 and November 2024 at a Level 1 Trauma Center, Cork University Hospital in the Republic of Ireland. The patients were monitored through a structured follow-up protocol that extended beyond six months, with the majority of patients being followed up for over 18 months. Follow-up assessments were conducted at defined intervals, including key evaluations at 3 and 6 months and at their final review. Clinical parameters such as pain, functional recovery, and radiological healing were considered. **Results**: No significant functional difference was observed at follow-up between the patients who sustained displaced fractures and those who presented with undisplaced fractures. Sixty percent of the patients remained pain-free from the 3-month postoperative follow-up, and the same percentage continued to be pain-free at the final follow-up. **Conclusions**: Cephalomedullary nailing is a safe option for the treatment of atypical femoral fractures. Patients with a bisphosphonate atypical femoral fracture should undergo bilateral screening and should be followed up for a longer period than the standard post-traumatic care intervals that are in place for typical femoral fractures.

## 1. Introduction

Osteoporosis is an important global health concern worldwide. It has been reported that 30% of women in the United States and Europe have osteoporosis [1]. Currently, it is estimated that 40% of postmenopausal women experience insufficiency fractures during their lifetime [2].

Bisphosphonates (BPs) are commonly prescribed for the treatment of osteoporosis; however, certain complications arise with long-term bisphosphonate therapy (>5 years) [3].

The complication highlighted in this study was atypical femoral fracture (AFF), which is a rare but significant complication associated with prolonged bisphosphonate therapy [4].

AFFs are often located in the subtrochanteric or femoral diaphyseal region and their onset is linked to a low-energy mechanism of injury and minimal trauma.

The pathognomonic radiological finding is a transverse or short oblique configuration of the fracture pattern, which can present as either an incomplete or complete pattern.

Histologically and pathophysiologically, AFFs result from severely suppressed bone remodeling due to chronic inhibition of osteoclast activity. This suppression leads to microdamage accumulation, compromised repairing mechanisms, and increasing cortical brittleness. The resulting stress concentration, which often appears on the lateral cortex, predisposes the femur to mechanical failure over time [5].

The orthopedic consensus identifies surgical intervention as the gold-standard treatment for atypical femoral fractures (AFFs), typically involving intramedullary nailing or cephalomedullary nailing (CMN), performed either unilaterally or prophylactically bilaterally [6,7].

Clinically, a notable proportion of patients report prodromal pain in the groin, thigh, or trochanteric area weeks to months before the occurrence of a complete fracture. In the study cohort, 60% of patients remained pain-free from the 3-month postoperative follow-up to the final review, while 40% of the patients included in the study reported localized pain, most frequently in the trochanteric bursa region. These symptoms may reflect underlying bony or soft tissue remodeling dysfunctions, consistent with the pathophysiology of AFFs.

The absolute risk of AFFs remains low but increases significantly with treatment duration. The literature reports that the risk of appearance of AFFs rises from 1.78 per 100,000 person-years after 2 years of bisphosphonate therapy to over 100 per 100,000 person-years after 8 years of continuous use [8]. It is also reported in the specialty literature that high adherence to long-term alendronate use (over 10 years) was associated with a lower risk of proximal femoral fractures overall but at the same time may elevate the risk of subtrochanteric and femoral shaft fractures. These findings underscore the complexity of long-term bisphosphonate use and the need for periodic reevaluation [9].

The complexity of these cases, along with the growing interest in AFF treatment, stems from the need for meticulous follow-up. This ensures proper healing during the course of recovery and helps prevent potential complications such as periprosthetic fractures or hardware failure.

This study aimed to evaluate the clinical outcomes, including radiological healing, functional recovery, and pain, in patients with atypical femoral fractures treated surgically with cephalomedullary nailing, using a structured follow-up protocol. Additionally, the study sought to highlight the importance of extended follow-up duration in the monitoring of AFF patients, given the complex patterns and long-term recovery involved in their treatment.

## 2. Materials and Methods

This single-center analysis was conducted on a mixed cohort comprising a total of 10 patients.

This retrospective study was conducted at Cork University Hospital (CUH) between June 2022 and April 2024, including patients who underwent surgical treatment for bisphosphonate-related atypical femoral fractures. Data collection and follow-up assessments were finalized by November 2024. Patients included in the study had undergone surgical fixation at least 6 months prior to the final data lock to ensure a minimum follow-up duration, with the majority of patients being followed up for over 18 months.

Inclusion and exclusion criteria can be found in the following table (Table 1):

Ten patients were included in this study. All ten patients with a history of prolonged bisphosphonate therapy for osteoporosis were included in the study. Collectively, these patients underwent 15 surgical procedures, as bilateral fixation was deemed necessary owing to the presence of AFFs or the identification of periosteal reactions on the contralateral side.

One surgical procedure was excluded from our study due to the nature of the injury. The respective AFF was represented by a periprosthetic AFF on total hip replacement, and because surgical fixation did not involve cephalomedullary nailing of the femur, it did not meet the inclusion criteria of the study.

All patients included in the study had a confirmed diagnosis of osteoporosis. Comorbidities were common among the cohort and included chronic conditions that may affect bone health or fracture healing. The patient characteristics and comorbidities can be found in the following table (Table 2).

The aim was to monitor patients for a follow-up period exceeding 6 months after surgical fixation with a CMN, with the majority of patients being followed up for more than 18 months after their initial surgery.

Follow-up was conducted in a manner that included the following criteria as key considerations: seeking fracture healing, functional outcomes, and medical management of the patients:

1. Radiological healing of AFFs;
2. Hardware integrity;
3. Continuation or discontinuation of bisphosphonate therapy after the occurrence of AFFs;
4. Treatment with alternative medication for osteoporosis after AFFs;
5. Fracture mobility scores (Table 3) [10].

6. Parker mobility scores (Table 4) [11]

Descriptive statistics were used to summarize demographic and clinical characteristics. The Shapiro–Wilk test was applied to assess the normality of continuous variables, such as age. Categorical variables were reported as frequencies and percentages, and continuous variables were expressed as means ± standard deviation, depending on distribution. Correlation analyses were performed using Pearson’s or Spearman’s rank correlation coefficient, as appropriate. Statistical analyses were performed using Microsoft Excel. A two-tailed *p* value < 0.05 was considered statistically significant.

## 3. Results

All 10 patients included in the study were Caucasian females and had been undergoing treatment for osteoporosis with bisphosphonates orally for periods of more than 5 years, ranging between 5 and 12 years.

**Figure 1:**
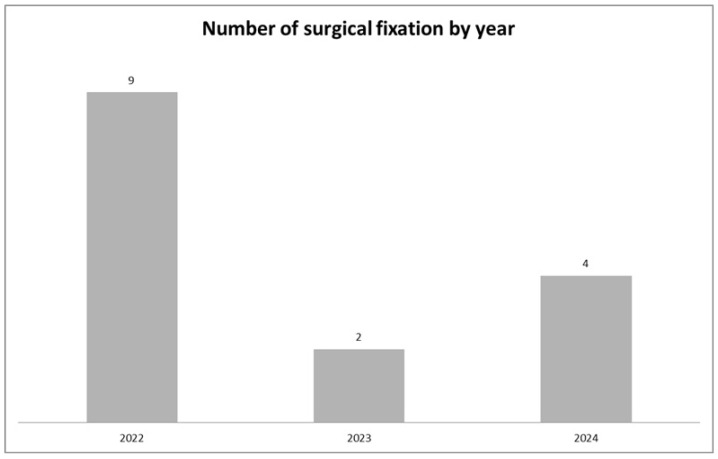
Number of surgical fixations by year.

The distribution of surgical procedures by year in which surgery took place is represented in the following figure (Figure 1).

**Figure 2:**
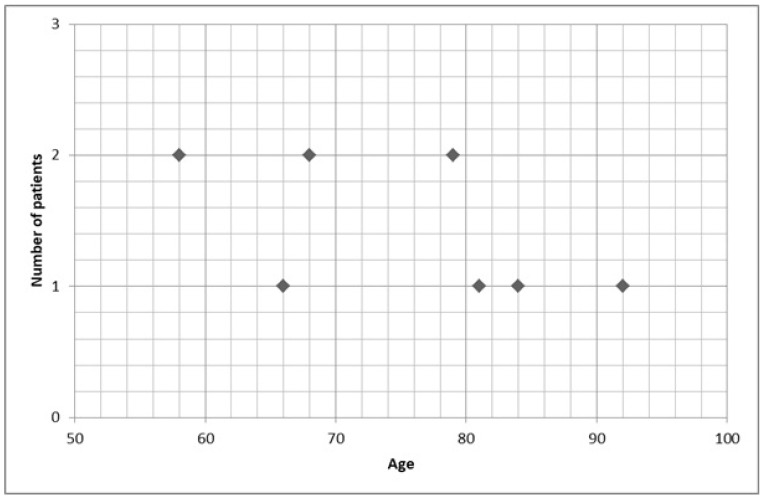
Distribution of patients by age.

The patients included in this study were aged between 58 years and 92 years, as shown in the following figure (Figure 2). The mean age of the patients included in the study was 73.3 years.

The age distribution was assessed for normality using the Shapiro–Wilk test and was found to be normally distributed (*p* = 0.527).

**Figure 3:**
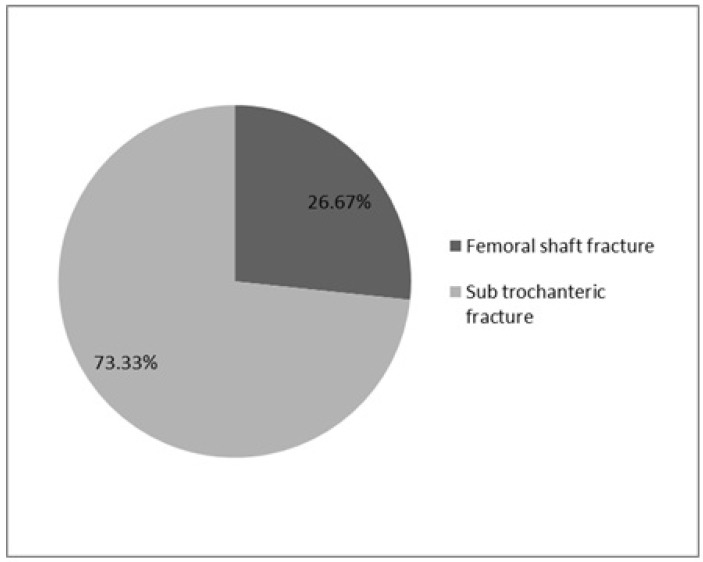
Distribution by fracture type.

AFFs were observed in both subtrochanteric and femoral shaft regions. Of these fractures, 73.33% were subtrochanteric and 26.67% occurred in the femoral shaft (Figure 3).

A total of 15 surgical procedures were performed across the 10 patients included in the study. Of these, five patients (50%) presented with bilateral atypical femoral fractures:

- Two patients underwent synchronous bilateral fixation, where contralateral lesions were identified on initial admission and treated within the same hospitalization.
- The remaining three patients underwent metachronous bilateral surgery, with contralateral lesions identified during outpatient follow-up based on prodromal symptoms or surveillance imaging.

The remaining five patients were treated for unilateral AFFs only.

**Figure 4:**
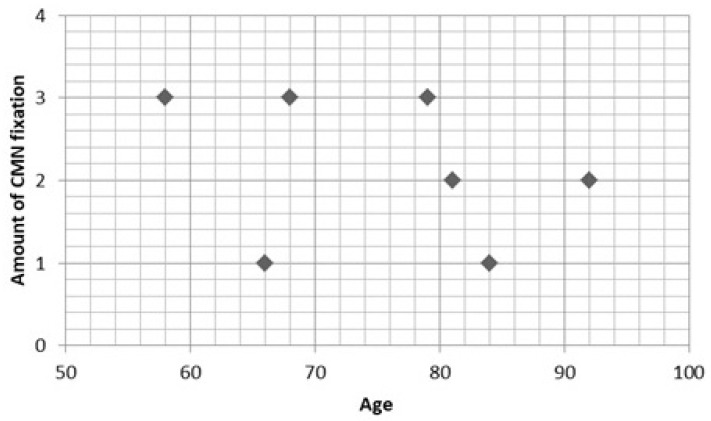
Distribution of surgical fixation by CMN in relation to patient’s age.

The distribution of surgeries according to age is shown in the following figure (Figure 4).

Analysis of the data using Pearson correlation revealed the following:

1. There was no significant difference between the age of the patients and the number of patients who developed AFFs (*p* = 0.190, *p* significant < 0.05).

2. There was no statistically significant difference between the age of the patients and the number of surgical fixations by CMN performed for AFFs (*p* = 0.498, *p* <0.05).

3. There was a strong statistical correlation between the number of patients and the number of surgical fixations by CMN in the sample of patients (*p* = 0.007, *p* < 0.05).

The above analysis demonstrates that bilateral AFFs were not associated with patient age. Additionally, bilateral fixation was necessary in half of the patients in our study because of the correlation between the number of patients and bilateral injuries, which were unaffected by patient age.

The patients included in the study were initially followed up according to the standard trauma protocol implemented at the CUH. All 10 patients were discharged from the fracture clinic of the Outpatient Department (OPD) in the CUH after assessment and were deemed clinically healed. The initial follow-up period before discharge ranged from 2 to 7 months postoperatively, with an average of 4.3 months before discharge from the OPD.

During the study period, the patients were recalled to the OPD at the CUH Orthopedic OPD for clinical and radiological assessment. During this secondary follow-up, the duration since surgical fixation ranged between 5.5 months and 31 months (over 2 years), with a mean follow-up time of 20.1 months, a median follow-up time of 18.5 months, and a standard deviation of 7.9 months.

While most patients were followed up for over 18 months, the lower limit of 5.5 months corresponds to one patient who had undergone surgery closer to the study period, thereby limiting the maximum available follow-up time within the study period. Despite the shorter duration, this patient had achieved radiological healing at the final review.

Notably, both the mean and median follow-up periods exceeded the initial targeted timeframe of 18 months, postoperatively.

Spearman’s rank correlation was used to explore the relationship between follow-up duration and patient age; this showed a strong positive correlation (ρ = 0.915, *p* < 0.0001).

**Figure 5:**
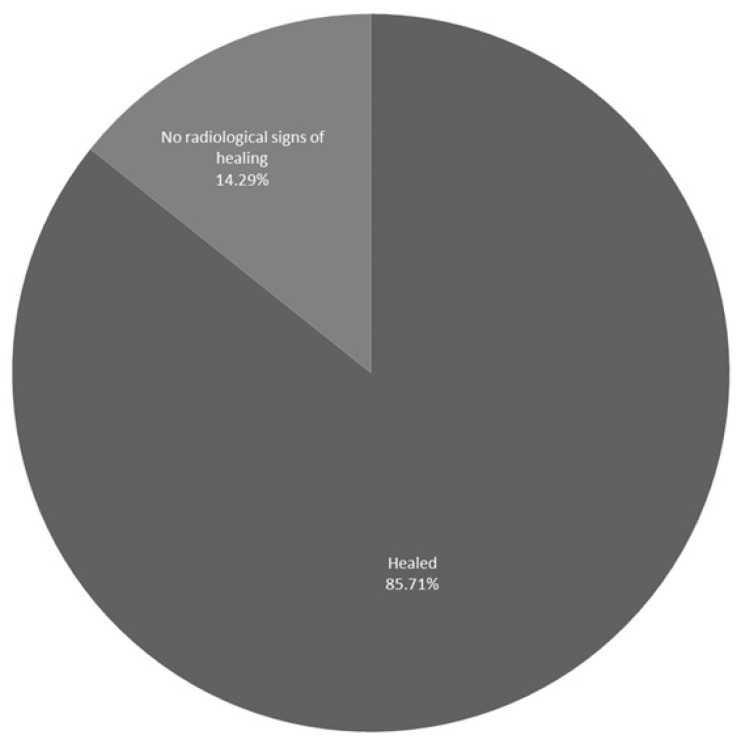
Healing rates of atypical fractures included in the study.

All 15 surgical procedures were followed up, 13 fractures had proven full radiological healing, and 2 of the atypical fractures did not heal from the time of surgery until their last follow-up assessment (Figure 5).

**Scheme 1:**
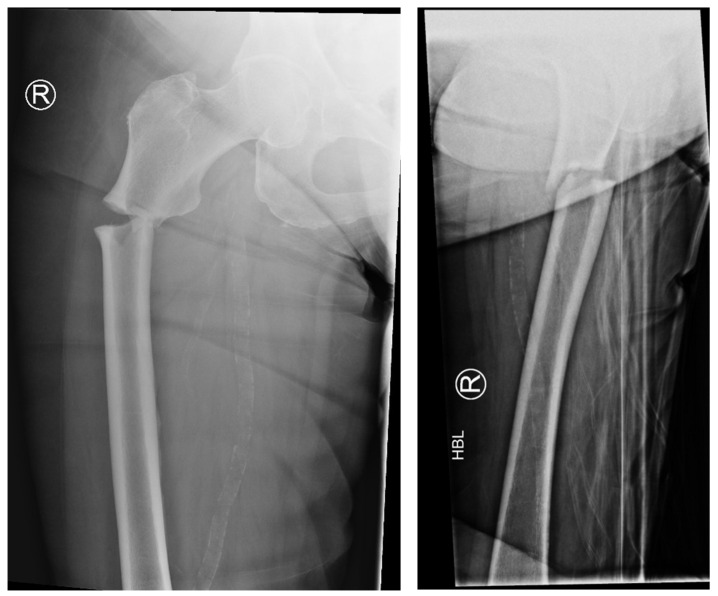
X-ray of right hip AP view (**left**) and lateral view of right proximal femur (**right**) of atypical subtrochanteric fracture—September 2023.

**Scheme 2:**
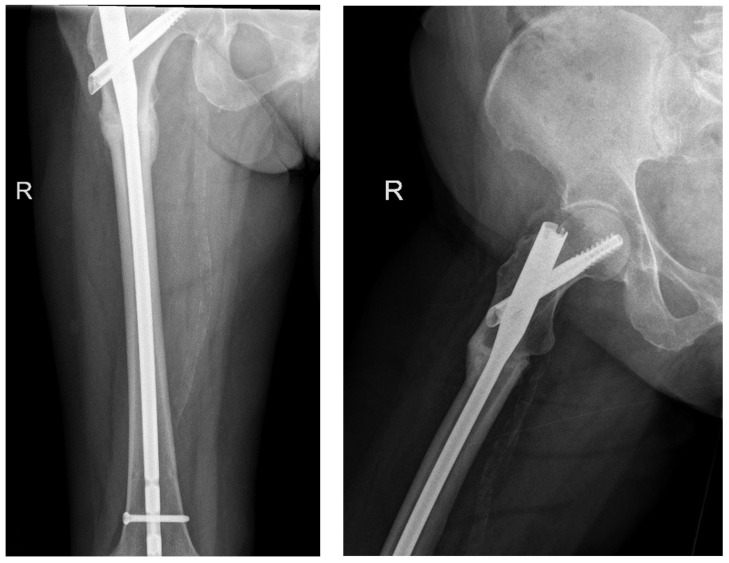
X-ray of right femur AP view (**left**) and lateral view of right hip (**right**) of atypical subtrochanteric fracture—September 2024 (radiological union).

**Scheme 3:**
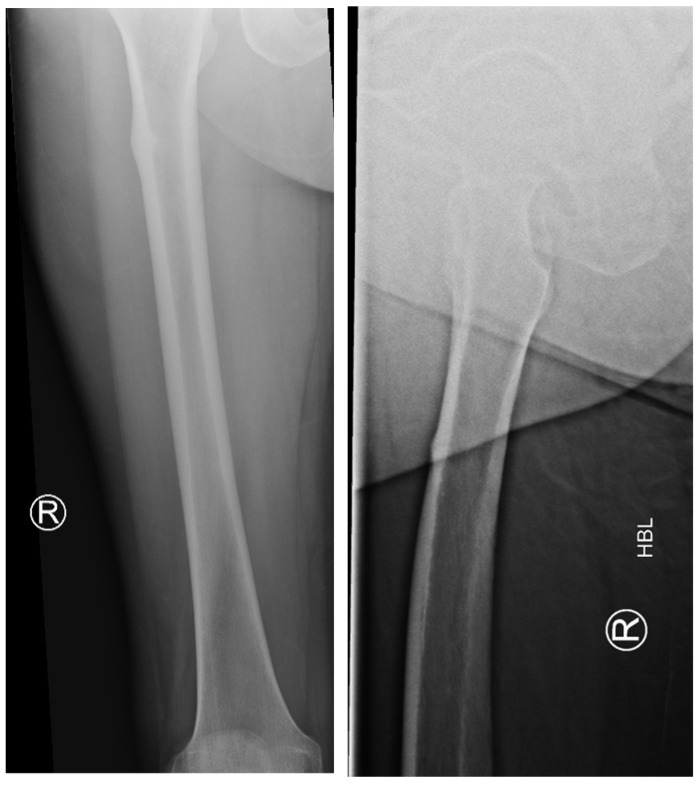
X-ray of right femur AP view (**left**) and lateral view (**right**) of atypical subtrochanteric stress fracture—July 2022.

**Scheme 4:**
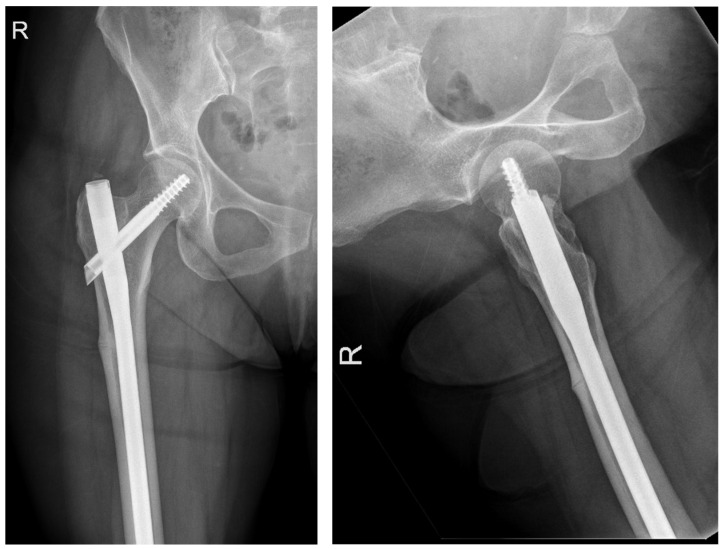
X-ray of right femur AP view (**left**) and lateral view (**right**) of atypical subtrochanteric stress fracture—September 2024 (no features of healing).

The following radiographs present the initial radiographs from the day of injury along with those obtained during the final OPD follow-up of the patient. The sequence presents radiographs demonstrating full radiological union (Scheme 1 and Scheme 2) and X-rays with no evidence of fracture healing at the final OPD follow-up (Scheme 3 and Scheme 4).

In the present study, there was no proven connection between radiological healing and discontinuation of bisphosphonate therapy or denosumab replacement therapy for osteoporosis.

The two fractures that showed no evidence of radiological healing at the last follow-up were atypical subtrochanteric stress fractures of the femur (unicortical fractures). There may be a potential relationship between fracture healing and fracture patterns. However, because the study included only two cases of non-healed fractures, both of which were similar in type, it is challenging to draw definitive conclusions. Notably, one patient did not receive osteoporotic treatment following the incident, whereas the other patient was undergoing treatment with denosumab for osteoporosis. Although it cannot be conclusively stated that the fracture type influences healing, this observation warrants further investigation in future studies.

No cases of hardware failure were reported during follow-up.

Considering the fracture mobility score, nine patients showed no change between the preoperative and postoperative scores, as all were mobilized freely without the use of aids. Only one patient experienced a reduction in mobility and required a single aid on the ipsilateral side fixed by CMN. This patient sustained a periprosthetic fracture (PPF) on the contralateral side, which necessitated multiple surgeries for repair and was the reason for the decreased mobility. In particular, the patient reported no pain or mobility issues on the side fixed with the CMN.

No significant functional difference was observed at follow-up between the patients who sustained displaced fractures and those who presented with undisplaced fractures.

All 10 patients included in the study discontinued bisphosphonate treatment once they were diagnosed with atypical femoral fractures. Postoperatively, 60% of the patients did not receive any alternative osteoporosis treatment following the discontinuation of bisphosphonate therapy. In contrast, 40% of patients were treated with denosumab as an alternative therapy for osteoporosis; alternative treatment was started after the surgical fixation of the AFFs. In all patients receiving denosumab, therapy was initiated postoperatively at varying intervals. While precise timing varied, treatment commencement occurred only after radiological signs of fracture healing had been established, generally no earlier than three months following surgical fixation; in all cases, the treatment had been started in the interval between three months and six months postoperatively.

**Figure 6:**
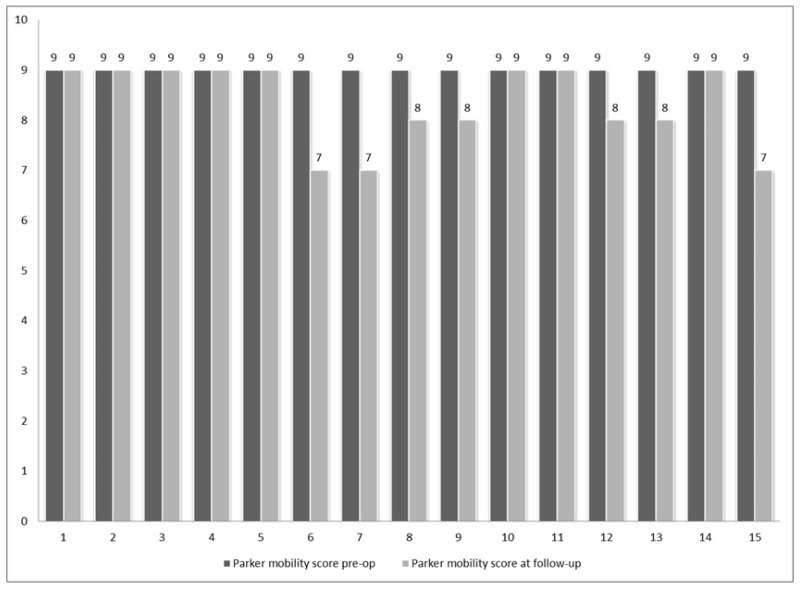
Representation of Parker mobility scores preoperatively and at follow-up.

Parker mobility scores were obtained for all patients included in this study, and the results showed that seven patients regained their baseline full independence (Parker mobility score was 9 both preoperatively and postoperatively at follow-up), which amounts to 70% of patients. One patient who presented with a preoperative Parker mobility score of 9 and underwent bilateral surgical fixation experienced a slight decrease in the score to 8, representing 10% of the patient cohort. Ultimately, two patients who required bilateral surgery—one with bilateral CMN of the femur and the other with a CMN on one side and revision surgery of a total hip replacement on the contralateral side—experienced a decrease in their Parker mobility score to a value of 7. Statistical analysis of the Parker mobility scores indicated that in our sample, bilateral involvement had a negative impact on regaining baseline mobility (*p* = 0.0133; *p* <0.05) (Figure 6).

## 4. Discussion

All patients in the study cohort were postmenopausal females, consistent with the known epidemiology of osteoporosis and AFFs. Estrogen deficiency is a critical factor in bone turnover reduction, potentially amplifying the effects of long-term bisphosphonate therapy. While the study reflects the typical sex distribution, future studies should investigate whether sex-related biological or pharmacological differences affect fracture patterns or fracture healing.

The presence of comorbidities such as cardiovascular disease, malignancy, autoimmune disorders, and nutritional deficiencies may influence fracture healing and postoperative recovery. Although the sample size was small, the variability in patient comorbid profiles underscores the need for individualized postoperative management. Notably, one patient in the cohort was receiving chronic corticosteroid therapy, which is known to adversely affect bone remodeling and healing capacity, but that patient had proven bone union on radiological follow-up. Future studies with larger sample sizes should stratify outcomes by comorbidity burden and steroid treatment exposure to better assess their impact on healing trajectories and functional outcomes.

Atypical femoral fractures of the subtrochanteric or diaphyseal areas of the femoral shaft have been reported in patients receiving bisphosphonates for osteoporosis treatment [12].

The literature has revealed a low absolute risk of AFFs, with ranges between 3.2 and 50 cases/100,000 person-years of exposure, with a significant influence of this range with prolonged duration of BPs (which is considered to be >3 years for intravenous BP treatment and >5 years for oral BP treatment). There are reports of a decrease in the risk of AFFs of up to 70% per year with discontinuation of treatment [13].

A Danish study previously found that there was no increase in the risk of femoral shaft or subtrochanteric fractures associated with high adherence to alendronate treatment compared to low adherence to alendronate treatment. The same study found that alendronate treatment for > 10 years was associated with a 30% lower risk of proximal femoral fractures (femoral neck and intertrochanteric fractures) [14].

A multicenter case–control study conducted in South Korea with a sample of 196 patients with AFFs found that the mean age of the patients with AFFs was 72 years, whereas the present study found that the mean age of the patients was 73.3 years [15].

While this study offers valuable insights into AFFs within the orthopedic department of a tertiary hospital in Ireland, several limitations must be acknowledged to effectively contextualize the findings.

This study involved a sample of 10 patients, limited to a single tertiary hospital’s orthopedic department. The relatively small sample size and specific patient population limit the generalizability of the results to other hospital departments or institutions with different patient demographics and clinical conditions. A smaller dataset introduces the potential for statistical variability, which may lead to an overestimation or underestimation of the AFF risk in patients undergoing bisphosphonate treatment. To enhance the robustness and reliability of future analyses, larger multicenter studies encompassing a more diverse patient population should be conducted.

This study’s findings were derived from a single hospital in Ireland, potentially limiting their applicability to other regions or healthcare settings. Future research should prioritize data collection from multiple hospitals across various regions to improve the external validity of the results and facilitate comparisons with global resistance trends.

An important limitation of this study is the lack of Bone Mineral Density data for the patients included. While all the patients had a clinical diagnosis of osteoporosis, and also a history of prolonged bisphosphonate therapy, individual Bone Mineral Density values were not available for the research and for review. This limits the ability to stratify patients based on osteoporosis severity or correlate Bone Mineral Density levels with fracture patterns, healing potential, or outcomes. Future prospective studies should incorporate baseline and follow-up Bone Mineral Density measurements to better assess these associations.

The present study discovered that 73.33% of fractures included were in the subtrochanteric region and only 26.67% were represented by femoral shaft AFFs, in contrast to another study conducted in Asia which reported 56% of AFFs at the level of the femoral shaft and only 44% in the subtrochanteric region [16]. This seems to corroborate the literature that femoral bowing in the Asian population represents an increased risk of AFFs [17].

Complete radiological union was achieved in 85.41% of the cases reported in the study, with a final follow-up mean of 18.5 months, and signs of healing as early as 6 weeks postoperatively in some of the cases included.

No impact on fracture healing was observed with the use of alternative osteoporosis medication. Forty percent of patients were treated with denosumab following discontinuation of bisphosphonate therapy, and it can be stated that within this patient sample, denosumab did not interfere with or delay radiological fracture healing. However, there are reports of recurrent incomplete AFFs and complete contralateral AFFs in patients treated with denosumab after the discontinuation of bisphosphonate therapy [18].

Currently, teriparatide therapy is considered an alternative treatment for AFFs; however, data suggest that although teriparatide reduces the risk of typical fragility fractures and might result in faster healing of surgically treated AFFs, there is insufficient evidence for teriparatide to be considered the gold standard in the treatment of incomplete AFFs [19].

The study reported no change in the fracture mobility score before and after the occurrence of AFFs, with full recovery of the baseline mobility. However, the study also reported a statistically significant decrease in the Parker mobility score between the samples of patients with unilateral lesions compared to those with bilateral lesions.

Even though all patients returned to baseline and did not require any crutches to mobilize, only 60% of our patients were pain-free; on the other hand, 40% of patients still presented with hip bursitis-associated pain, which might also be caused by their underlying chronic changes. There have been reports of patients being pain-free in smaller samples of patients, but all the included patients in that study had bilateral AFFs and bilateral surgical fixation by CMN [20].

Prodromal pain is an important characteristic in the clinical evaluation of patients treated with bisphosphonates and can be used as a red flag in management; this symptom is also included in the National Osteoporosis Guideline Group (NOGG) Guideline. The literature reports prodromal pain in the groin, hip area, and lateral or anterior thigh, ranging from 32% to 76%, in patients with incomplete or developing AFFs. Prodromal pain can occur months before AFF onset and is seemingly correlated with periosteal or endosteal reactions [21].

To strengthen the external validity of our findings, our results were compared with other studies focusing on AFF healing, function, and pharmacological management.

In the present study, a radiological union rate of 85.7% was achieved across 15 atypical femoral fractures treated with CMN, which compares favorably with data from prior publications. For instance, Prasarn et al. reported a similar union rate of 86% in their cohort of 22 surgically treated AFFs, though they noted a 14% rate of delayed union in patients who continued bisphosphonate therapy postoperatively. This aligns with our observation that fracture pattern, rather than pharmacologic management alone, may influence healing timelines [22].

Regarding adjuvant anabolic therapies, while none of the patients in the present study cohort were treated with teriparatide, its emerging role in enhancing healing has been documented. In a matched case–control study, Choi et al. demonstrated that the use of teriparatide was associated with a significantly shorter median healing time (4.5 months vs. 8.6 months) and fewer reoperations. Although our sample size was small, the two cases of non-union—both involving stress fractures—raise questions about whether adjuvant teriparatide treatment could benefit selected patients with impaired healing [23].

The bilateral nature of AFFs was another important aspect of our study, with 50% of patients requiring bilateral surgical fixation. This observation is consistent with the study of Capeci and Tejwani, who found a 41% incidence rate of bilateral AFFs in their cohort, often diagnosed metachronously. Their recommendation for systematic contralateral imaging at the time of diagnosis reinforces our protocol of bilateral screening, especially in patients with prodromal pain or suggestive imaging findings [24].

The impact of corticosteroid therapy on bone health and fracture healing is well documented. Although only one patient in our study was on chronic corticosteroids and achieved complete union, this finding contrasts with a large population-based study by Lo et al., which identified corticosteroid exposure as a risk factor for both bilateral AFFs and delayed healing. This discrepancy may be attributable to sample size, and larger studies are needed to stratify healing outcomes based on cumulative steroid exposure [25].

From a functional perspective, our use of the Parker mobility score revealed that 70% of patients regained baseline ambulatory function postoperatively. This is highly consistent with the findings of Li et al., who reported that 64% of AFF patients treated surgically recovered their pre-fracture mobility [26]. Notably, both studies identified bilateral fixation as a predictor of decreased postoperative mobility, emphasizing the importance of individualized rehabilitation and follow-up in this subgroup.

Collectively, these comparisons demonstrate that our results align well with the broader AFF literature, especially with regard to healing potential, bilateral involvement, and the influence of patient-specific variables such as pharmacologic therapy and comorbidities. The consistency between our findings and prior reports supports the external validity of our observations and underlines the need for continued research into targeted interventions for patients with atypical femoral fractures.

Prevention of AFFs has become an increasingly important focus in the management of long-term osteoporosis therapy. Current best practices emphasize individualized risk-benefit assessments for patients undergoing extended bisphosphonate treatment, particularly beyond a period of five years. One cornerstone of prevention is the implementation of a drug holiday strategy. This involves pausing bisphosphonate therapy after 3–5 years in low-risk individuals or after 10 years in the case of high-risk patients, thereby reducing the risk of oversuppression of bone turnover and microdamage accumulation. Regular monitoring of prodromal symptoms, such as thigh or groin pain and radiographic screening of the contralateral femur, is an essential component of the early detection of pathological changes. Additionally, periodic reevaluation of Bone Mineral Density using dual-energy X-ray absorptiometry scans and assessment of clinical fracture risks via tools such as the fracture risk assessment tool can guide ongoing treatment decisions [27]

In recent years, attention has also turned to sequential therapy, where patients are transitioned from bisphosphonate therapy to anabolic agents such as teriparatide or romosozumab. Teriparatide has shown promise in improving fracture healing and potentially reversing the suppressed bone remodeling associated with long-term antiresorptive therapy. While not yet considered standard for primary prevention of AFFs, these agents may play a crucial role in secondary prevention of AFFs or as an alternative treatment for patients that have already sustained AFFs. Further, patient education about warning signs and adherence to national guidelines, such as those from the NOGG or the American Society for Bone and Mineral Research (ASBMR), is vital in minimizing risk and optimizing long-term outcomes [28].

The fracture pattern of atypical femoral fractures might be connected to full radiological union of the fracture; however, follow-up and inclusion of larger patient samples are required to conclusively determine whether atypical stress (unicortical) fractures in the subtrochanteric region are least likely to attain complete radiological union [29].

Adjuvant medical therapies should be considered in the future, and promising research is being conducted on teriparatide administration to aid fracture healing in patients with delayed union or non-union who have a history of bisphosphonate therapy [30].

All patients in our cohort were menopausal females. While this reflects the predominance of osteoporosis among women, the absence of male patients in our study is a notable limitation. The increasing recognition of osteoporosis in men, coupled with evidence suggesting potentially poorer outcomes in male patients with atypical femoral fractures, underscores the need for sex-specific research in this area. Future studies should aim to include male patients to better understand sex-related differences in fracture patterns and outcomes [31].

Prodromal pain should be considered a red flag for the proper screening of patients undergoing bisphosphonate treatment; however, it should be noted that it is not pathognomonic.

Full adherence to the NOGG should be aimed for given the complexity of these injuries, which should be managed using a multimodal approach with proper radiological monitoring, functional assessment, and pharmacological adjustments. A multidisciplinary approach seems suitable for managing these injuries, and close collaboration between orthopedic surgeons, rehabilitation specialists, endocrinologists, and general practitioners should be established as the standard of care for patients undergoing bisphosphonate treatment.

Patients with a bisphosphonate atypical femoral fracture should undergo bilateral screening and should be followed up for a longer period than the standard post-traumatic care intervals that are in place for typical femoral fractures.

## 5. Conclusions

This study supports cephalomedullary nailing as a safe and effective surgical approach for the management of bisphosphonate-related atypical femoral fractures, with the current study demonstrating a radiological healing rate of 85.71% and no hardware failures during follow-up. Although the sample size was limited, the findings support existing literature regarding functional recovery and healing outcomes following CMN fixation. Additionally, the frequency of bilateral involvement observed in the current cohort reinforces the need for routine contralateral femoral screening and long-term follow-up in patients with AFFs. These results highlight the importance of individualized postoperative care and interdisciplinary collaboration in managing this complex patient population.

## Figures and Tables

**Table 1:** Inclusion and exclusion criteria.

Inclusion Criteria	Exclusion Criteria
Patients aged over 18 years	Periprosthetic femoral fractures (involving hip prosthesis
Confirmed diagnosis of AFFs according to American Society for Bone and Mineral Research (ASBMR) criteria	Pathological fractures from primary/metastatic malignancies or non-osteoporotic bone disorders
History of oral bisphosphonate use for over 5 years prior to the fracture	Follow-up less than 6-month threshold
Surgical treatment of AFF by cephalomedullary nailing	
Surgical procedure between June 2022 and April 2024	
Minimum follow-up of 6 months at the time of final data collection	

**Table 2:** Patient characteristics and comorbidities.

Patient	Sex	Comorbidities	Corticosteroid Use	History of Malignancy
**1**	Female	Osteoporosis	No	No
**2**	Female	Osteoporosis Vitamin B12 deficiency	No	No
**3**	Female	Osteoporosis Rheumatoid arthritis Hypertension Iron deficiency	Yes	No
**4**	Female	Osteoporosis Hypertension Ischemic heart disease	No	No
**5**	Female	Osteoporosis Pulmonary neoplasm Breast neoplasm Appendectomy Hysterectomy Small bowel obstruction	No	Yes (pulmonary and breast neoplasm)
**6**	Female	Osteoporosis	No	No
**7**	Female	Osteoporosis Melanoma	No	Yes (melanoma)
**8**	Female	Osteoporosis	No	No
**9**	Female	Osteoporosis	No	No
**10**	Female	Osteoporosis	No	No

**Table 3:** Fracture mobility score.

Fracture Mobility Score—Preoperatively	Fracture Mobility Score Final Follow-Up
Freely mobile without aids	Freely mobile without aids
Mobile outdoors with one aid	Mobile outdoors with one aid
Mobile outdoors with two aids or frame	Mobile outdoors with two aids or frame
Some indoor mobility but never going outside without help	Some indoor mobility but never going outside without help
No functional mobility (no use of lower limbs)	No functional mobility (no use of lower limbs)
Unknown (patient cannot specify)	Unknown (patient cannot specify)

**Table 4:** Parker mobility score.

Parker Mobility Score—Preoperatively	Parker Mobility Score—Final Follow-Up
Able to get about the house No difficulty (3 pts) With an aid (2 pts) With help from another person (1 pts) Not at all (0 pts)	Able to get about the house No difficulty (3 pts) With an aid (2 pts) With help from another person (1 pts) Not at all (0 pts)
Able to get out of the house No difficulty (3 pts) With an aid (2 pts) With help from another person (1 pts) Not at all (0 pts)	Able to get out of the house No difficulty (3 pts) With an aid (2 pts) With help from another person (1 pts) Not at all (0 pts)
Able to go shopping No difficulty (3 pts) With an aid (2 pts) With help from another person (1 pts) Not at all (0 pts)	Able to go shopping No difficulty (3 pts) With an aid (2 pts) With help from another person (1 pts) Not at all (0 pts)
Total (0–9 pts) =	Total (0–9 pts) =

## Data Availability

Data are contained within the article or Supplementary Materials and the original contributions presented in this study are included in the article. Further inquiries can be directed to the corresponding authors.

## Supplementary Materials

The following supporting information can be downloaded at https://www.mdpi.com/article/10.3390/jcm14082858/s1. Consent form and Information Leaflet.

## Abbreviations

The following abbreviations are used in this manuscript:

AFFs	Atypical femoral fractures
BPs	Bisphosphonates
CMN	Cephalomedullary nail
*p*	*p* value
OPD	Outpatient Department
CUH	Cork University Hospital
PPF	Periprosthetic fracture
NOGG	National Osteoporosis Guideline Group
ASBMR	American Society for Bone and Mineral Research

## References

[1] Cooper C., Campion G., Melton L.J. (1992). Hip fractures in the elderly: A world-wide projection. Osteoporos. Int..

[2] Reginster J.Y., Burlet N. (2006). Osteoporosis: A still increasing prevalence. Bone.

[3] van den Bergh J., van Geel T., Geusens P. (2012). Osteoporosis, frailty and fracture: Implications for case finding and therapy. Nat. Rev. Rheumatol..

[4] Bubbear J.S. (2016). Atypical Femur Fractures in Patients Treated with Bisphosphonates: Identification, Management, and Prevention. Rambam Maimonides. Med. J..

[5] Dell R.M., Adams A.L., Greene D.F., Funahashi T.T., Silverman S.L., Eisemon E.O., Zhou H., Burchette R.J., Ott S.M. (2012). Incidence of atypical nontraumatic diaphyseal fractures of the femur. J. Bone Miner. Res..

[6] Shane E., Burr D., Ebeling P.R., Abrahamsen B., A Adler R., Brown T.D., Cheung A.M., Cosman F., Curtis J.R., Dell R. (2010). Atypical subtrochanteric and diaphyseal femoral fractures: Report of a task force of the American Society for Bone and Mineral Research. J. Bone Miner. Res..

[7] Shane E., Burr D., Abrahamsen B., Adler R.A., Brown T.D., Cheung A.M., Cosman F., Curtis J.R., Dell R., Dempster D.W. (2014). Atypical subtrochanteric and diaphyseal femoral fractures: Second report of a task force of the American Society for Bone and Mineral Research. J. Bone Miner. Res..

[8] Meier R.P., Perneger T.V., Stern R., Rizzoli R., Peter R.E. (2012). Increasing occurrence of atypical femoral fractures associated with bisphosphonate use. Arch. Intern. Med..

[9] Black D.M., Kelly M.P., Genant H.K., Palermo L., Eastell R., Bucci-Rechtweg C., Cauley J., Leung P.C., Boonen S., Santora A. (2010). Bisphosphonates and fractures of the subtrochanteric or diaphyseal femur. N. Engl. J. Med..

[10] Voeten S.C., Nijmeijer W.S., Vermeer M., Schipper I.B., Hegeman J., De Klerk G., Stevens C., Snoek J., Luning H., Van der Velde D. (2020). Validation of the Fracture Mobility Score against the Parker Mobility Score in hip fracture patients. Injury.

[11] Parker M.J., Palmer C.R. (1993). A new mobility score for predicting mortality after hip fracture. J. Bone Joint Surg. Br..

[12] (2024). National Osteoporosis Guideline Group (NOGG) UK, Clinical Guideline for the Prevention and Treatment of Osteoporosis. https://www.nogg.org.uk/full-guideline.

[13] Silverman S., Kupperman E., Bukata S. (2018). Bisphosphonate-related atypical femoral fracture: Managing a rare but serious complication. Clevel. Clin. J. Med..

[14] Abrahamsen B., Eiken P., Prieto-Alhambra D., Eastell R. (2016). Risk of hip, subtrochanteric, and femoral shaft fractures among mid and long term users of alendronate: Nationwide cohort and nested case-control study. Br. Med. J..

[15] Lim S.-J., Yeo I., Yoon P.-W., Yoo J., Rhyu K.-H., Han S.-B., Lee W.-S., Song J.-H., Min B.-W., Park Y.-S. (2018). Incidence, risk factors, and fracture healing of atypical femoral fractures: A multicenter case-control study. Osteoporos. Int..

[16] Chen L.P., Chang T.K., Huang T.Y., Kwok T.G., Lu Y.C. (2014). The correlation between lateral bowing angle of the femur and the location of atypical femur fractures. Calcif. Tissue Int..

[17] van de Laarschot D.M., McKenna M.J., Abrahamsen B., Langdahl B., Cohen-Solal M., Guañabens N., Eastell R., Ralston S.H., Zillikens M.C. (2020). Medical Management of Patients after Atypical Femur Fractures: A Systematic Review and Recommendations from the European Calcified Tissue Society. J. Clin. Endocrinol. Metab..

[18] Hamahashi K., Noguchi T., Uchiyama Y., Sato M., Watanabe M. (2020). Clinical Features and Outcomes of Bilateral Atypical Femoral Fractures. Case Rep. Orthop..

[19] Bogdan Y., Einhorn T.A., Silverman S.L., Abrahamsen B. (2016). Clinical presentation of atypical femur fractures. The Duration and Safety of Osteoporosis Treatment.

[20] Egol K.A., Park J.H., Rosenberg Z.S., Peck V., Tejwani N.C. (2014). Healing delayed but generally reliable after bisphosphonate-associated complete femur fractures treated with IM nails. Clin. Orthop. Relat. Res..

[21] Schilcher J., Koeppen V., Aspenberg P., Michaëlsson K. (2015). Risk of atypical femoral fracture during and after bisphosphonate use. Acta Orthop..

[22] Prasarn M.L., Ahn J., Helfet D.L., Lane J.M., Lorich D.G. (2012). Bisphosphonate-associated femur fractures have high complication rates with operative fixation. Clin. Orthop. Relat. Res..

[23] Choi S.Y., Yoon D., Kim K.M., Kim S.J., Kim H.Y., Kim J.W., Park J.H. (2024). Adjunctive recombinant human parathyroid hormone agents for the treatment of medication-related osteonecrosis of the jaw: A report of three cases. J. Korean Assoc. Oral. Maxillofac. Surg..

[24] Capeci C.M., Tejwani N.C. (2009). Bilateral low-energy simultaneous or sequential femoral fractures in patients on long-term alendronate therapy. J. Bone Joint Surg. Am..

[25] Lo J.C., O’Ryan F.S., Gordon N.P., Yang J., Hui R.L., Martin D., Hutchinson M., Lathon P.V., Sanchez G., Silver P. (2010). Prevalence of osteonecrosis of the jaw in patients with oral bisphosphonate exposure. J. Oral. Maxillofac. Surg..

[26] Li Y.Y., Gao L.J., Zhang Y.X., Liu S.J., Cheng S., Liu Y.P., Jia C.X. (2020). Bisphosphonates and risk of cancers: A systematic review and meta-analysis. Br. J. Cancer.

[27] Black D.M., Rosen C.J. (2016). Clinical Practice. Postmenopausal Osteoporosis. N. Engl. J. Med..

[28] Chincholi A.H., Cooper A.R., Mullally J.A. (2024). Treatment of Bisphosphonate-Associated Atypical Femur Fracture with a Combination of Teriparatide and a Novel Surgical Technique. AACE Clin. Case Rep..

[29] Yoon B.H., Kim M., Roh Y.H. (2024). Does the Nonunion Rate of Atypical Femoral Fractures Differ According to Fracture Site?: A Meta-Analysis. Clin. Orthop. Surg..

[30] Greenspan S.L., Vujevich K., Britton C., Herradura A., Gruen G., Tarkin I., Siska P., Hamlin B., Perera S. (2018). Teriparatide for treatment of patients with bisphosphonate-associated atypical fracture of the femur. Osteoporos. Int..

[31] Adler R.A., Feingold K.R., Ahmed S.F., Anawalt B. (2000). Osteoporosis in Men. Endotext.

