# Supplementary material for: Management Concepts of Bisphosphonate-Related Atypical Femoral Fractures
Source: J Clin Med. 2025 Apr 21;14(8):2858. doi: 10.3390/jcm14082858 (PMC12027697; doi:10.3390/jcm14082858)
Supplement: Supplementary file 1 [file jcm-14-02858-s001.zip › Consent form.pdf]

PATIENT/PARTICIPANT CONSENT FORM

**Study title: Management concepts of bisphosphonate related atypical femoral fractures**

I have read and understood the Information Leaflet about this research project. The information has been fully explained to me and I have been able to ask questions, all of which have been answered to my satisfaction.	**Yes**	**No**
I understand that I don't have to take part in this study and that I can opt out at any time. I understand that I don't have to give a reason for opting out and I understand that opting out won't affect my future medical care.	**Yes**	**No**
I give permission for researchers to look at my medical records to get information. I have been assured that information about me will be kept private and confidential.	**Yes**	**No**
I have been given a copy of the Information Leaflet and this completed consent form for my records.	**Yes**	**No**
I give informed explicit consent to have my data processed as part of this research study.	**Yes**	**No**
I consent that the functional scores and radiological findings can be used for the publishing of this research paper	**Yes**	**No**
I consent to be contacted by researchers as part of this research study.	**Yes**	**No**
I consent to be re-contacted by researchers about possible future research unrelated to the current study for which I may be eligible.	**Yes**	**No**

| |
____
Patient/Participant Name (Block Capitals) | Patient/Participant Signature | Date

--- ---
Legal Representative/Guardian Name   Legal Representative/Guardian Signature   Date

To be completed by the Principal Investigator or nominee.

I, the undersigned, have taken the time to fully explain to the above patient the nature and purpose of this study in a way that they could understand. I have explained the risks involved as well as the possible benefits. I have invited them to ask questions on any aspect of the study that concerned them.

Liviu-Coriolan Misca | Orthopaedic Registrar | |

Name (Block Capitals) | Qualifications | Signature | Date
