# Supplementary material for: Management Concepts of Bisphosphonate-Related Atypical Femoral Fractures
Source: J Clin Med. 2025 Apr 21;14(8):2858. doi: 10.3390/jcm14082858 (PMC12027697; doi:10.3390/jcm14082858)
Supplement: Supplementary file 1 [file jcm-14-02858-s001.zip › Information Leaflet.pdf]

Patient information leaflet

Study title: Management concepts of bisphosphonate related atypical femoral fractures

You are being invited to take part in a research study.

However, before you decide whether or not to take part, it is important that you fully understand what the research is about and what you will be asked to do.

It is important that you read the following information in order to make an informed decision and if you have any questions about any aspects of the study that are not clear to you do not hesitate to ask me.

Please make sure that you are satisfied before you decide to take part or not.

This study aims to take in account radiological and functional outcomes of the surgical treatment of which you have benefited previously for the femoral atypical fracture you have suffered in relation with your bisphosphonate medical treatment for osteoporosis.

The study is retrospective and will look for information in regards to:

- Pre and postoperative function using the Fracture Mobility score and Parker Mobility score
- Radiological healing of the fracture at set time follow-up
- Continuation or intrerruption of bisphosphonate therapy after injury

During the study you will have follow-up for radiological healing and functional scoring at certain set times.

There are no risks associated with this study and the current study does not interfere with your treatment in any way.

The potential benefit of this study is that you will be followed up for a longer, than typical, period in the Orthopedic Outpatient Department of CUH and if there are any issues that arise in regards to your function they can be addressed at the time of your Orthopedic review.

At the end of this study the researchers will publish a paper on the functional and radiological findings.

No personal data will be made public or used for publishing. And all data collected will be done safely under GDPR.

Publishing will include age categories, sex and fracture type.

Radiological proof of healing/non-union might be used for publishing this current paper.

Data collected will be kept confidential under password protection and the signed consents will be digitalized and held for a period of 5 years. Afterwards the hard copies will be shredded to ensure confidentiality and the digitalized version will be kept under password protection.

Thank you for your time and consideration of this invitation!
